# Baseline symptom severity and efficacy of Silexan in patients with anxiety disorders: A symptom-based, patient-level analysis of randomized, placebo-controlled trials

**DOI:** 10.1192/j.eurpsy.2024.16

**Published:** 2024-03-01

**Authors:** Markus Dold, Hans-Jürgen Möller, Hans-Peter Volz, Erich Seifritz, Sandra Schläfke, Lucie Bartova, Siegfried Kasper

**Affiliations:** 1 Department of Psychiatry and Psychotherapy, Medical University of Vienna, Vienna, Austria; 2 Department of Psychiatry and Psychotherapy, Ludwig Maximilian University, Munich, Germany; 3 Hospital for Psychiatry, Psychotherapy and Psychosomatic Medicine Schloss Werneck, Werneck, Germany; 4 Department of Psychiatry, Psychotherapy and Psychosomatics, Psychiatric Hospital, University of Zürich, Switzerland; 5 Dr. Willmar Schwabe GmbH & Co. KG, Karlsruhe, Germany; 6 Center for Brain Research, Medical University of Vienna, Vienna, Austria

**Keywords:** anxiety disorders, efficacy, lavender, severity of illness, Silexan

## Abstract

The influence of baseline severity on the efficacy of Silexan, a proprietary essential oil from *Lavandula angustifolia*, in anxiety disorders has not been investigated in a pooled dataset. We report on an individual patient data analysis of all five double-blind, randomized, placebo-controlled trials with Silexan in anxiety disorders. Eligible participants received Silexan 80 mg/d or placebo for 10 weeks. Analyses were based on the Hamilton Anxiety Rating Scale (HAMA), its psychic and somatic anxiety subscores, and the Clinical Global Impressions (CGI) scale. To correlate baseline severity with outcome, patients were segregated into mild, moderate, and severe cases. Altogether 1,172 patients (Silexan, n = 587; placebo, n = 585) were analyzed. For the HAMA total score, we found a significant association between the score at baseline and the treatment effect of Silexan versus placebo at week 10 (*p* < 0.001). HAMA items from the somatic domain scored lower at baseline and showed less improvement than items from the psychic domain, particularly in patients with mild or moderate baseline symptoms. For CGI item 2 (global improvement), significant efficacy favoring Silexan were observed in mild, moderate, and severe baseline symptom severity. Although significant improvements were found for all subsets, the more severe the initial symptoms, the greater the treatment effects documented by the HAMA. Overall this analysis confirms that Silexan is an effective treatment option in early or mild stages of anxiety disorder. Given its favorable safety profile, Silexan can thus fill a therapeutic gap in the treatment of (subsyndromal) anxiety disorders.

## Background

Anxiety disorders are the most common mental illnesses, with an age-standardized prevalence of approximately 4,000 per 100,000 people globally and of approximately 5,000 per 100,000 people in “high-income” countries in 2019 [1]. Even though anxiety disorders are often associated with a high disease burden [2], only a minority of affected patients receive appropriate therapeutic care [3–5].

Selective serotonin reuptake inhibitors (SSRIs) and serotonin and noradrenaline reuptake inhibitors (SNRIs) are recommended as first-line psychopharmacotherapy for anxiety disorders [e.g., 6–11]. While there is differentiating evidence regarding the efficacy of SSRIs/SNRIs in episodes of different degrees of severity in the treatment of depression [12, 13], the association between initial severity and treatment efficacy appears to be even less well understood. With regard to anxiety disorders, several meta-analyses have found no association between initial severity of anxiety and the efficacy of anxiolytic treatment [14–17]. However, a meta-analysis based on individual patient data of patients with generalized anxiety disorder (GAD) and panic disorder showed larger benefits of treatment in more severe cases, but only fewer benefits at low initial severity [18].

The inconsistency of the results regarding the association between the initial severity of anxiety and treatment efficacy may be at least partly attributable to methodological issues. Firstly, meta-analyses published to date have used a summary measure of a clinical scale, mainly the total score of the Hamilton Anxiety Rating Scale (HAMA) [19], for characterizing both initial severity and treatment efficacy. It can, however, not be excluded that patients with lower initial HAMA total scores may suffer from a qualitatively different condition than those with higher scores, that is, pre-treatment total score differences may be attributable to subsets of HAMA items, which may not be apparent when relying on the total score alone. Secondly, as already mentioned, most meta-analyses have been performed on aggregated trial-level data, not on individual patient-level data. It has been demonstrated, however, that the interpretation of trial-level meta-analysis results may bear relevant difficulties when the predictor (i.e., average initial symptom severity) is a patient-level characteristic that has been averaged over study patients [20]. This may mislead researchers into observing a trial-level association that is not attributable to the patient level or vice versa [21].

The ongoing debate about the association between the initial severity of the disease and the efficacy of anxiolytic/antidepressant psychopharmacotherapy may contribute to the reluctance of practitioners to provide medication to less severe cases, also considering the potential risks and side effects still associated with newer-generation antidepressants [e.g., 2, 22, 23]. This is a meaningful challenge in view of the fact that undertreatment of anxiety has been reported particularly for patients suffering from a subsyndromal presentation of the disorder [24, 25]. It is critical to recognize that for these patients the symptom burden and suffering may not differ substantially, if at all, from those patients who meet all ICD or DSM criteria for GAD [26].

Therefore, further insights are relevant and important for providing appropriate treatment to patients suffering from anxiety disorders at all severity levels.

In this paper, we report on a patient-level, item-based, post-hoc analysis of all double-blind, placebo-controlled trials investigating the efficacy of Silexan^1^ – an essential oil for oral administration that is manufactured from *Lavandula angustifolia* flowers and registered as a medicinal product – in patients suffering from anxiety disorders. Characterizations of the pharmacological profile of Silexan have been published elsewhere [27, 28]. Meta-analyses investigating the efficacy of Silexan in patients with anxiety disorders have been presented by Generoso et al. [29], Yap et al. [30], and others. Moreover, a meta-analysis by Möller et al. [25] has demonstrated that Silexan is efficacious in patients with subsyndromal anxiety.

## Methods

### Data acquisition

We obtained patient-level data from five clinical trials [31–35] (Trials A–E), representing all randomized, placebo-controlled trials – sponsored by Dr. Willmar Schwabe GmbH & Co. KG (Karlsruhe, Germany), manufacturer of Silexan – performed on patients with anxiety disorders and completed by October 2021.

In addition, we systematically screened PubMed/Medline for further relevant randomized, placebo-controlled trials using the search terms “Silexan” and “lavender oil.” However, no further relevant studies could be identified.

The trials included into the analysis were performed according to essentially similar protocols (Table 1). Participants were male or female outpatients, between 18 and 65 years, suffering from a subsyndromal anxiety (three studies) disorder or GAD (two studies), who were recruited in psychiatric or/and general practices. Eligible patients were randomized to receive 10 weeks’ treatment with 1 × 80 mg/d Silexan or a matching placebo. Further details of the dataset used for this study have been described in Bartova et al. [36].

**Table 1.** Main study design characteristics, patient inclusion criteria, and number of patients in the full analysis set

Trial	A [31]	B [32]	C [33]	D [35]	E [34]
Design	Double-blind, randomized, placebo-controlled, multicenter, parallel group				
Diagnosis for inclusion	Anxiety disorder not otherwise specified (DSM‑IV 300.00; ICD-10 F41.9)	Restlessness and sleep disturbances (ICD‑10 R45.1)	Mixed anxiety and depressive disorder (ICD‑10 F41.2)	Generalized anxiety disorder (DSM IV 300.02, also corresponding to DSM-5 criteria; ICD 10 F41.1)	
Specific inclusion criteria	HAMA total score ≥18 points; HAMA items “anxious mood” and “insomnia” ≥2 points	HAMA total score ≥18 points; HAMA items “tension” and “insomnia” ≥2 points	HAMA total score ≥18 points; HAMA item “anxious mood” ≥2 points	HAMA total score ≥18 points; HAMA items “anxious mood” and “tension” ≥2 points; CAS total score ≥9 points	HAMA total score ≥18 points; HAMA items “anxious mood” and “tension” ≥2 points; HAMA subscore “psychic anxiety” ≤21 points; CAS total score ≥9 points
Interventions	1 × 80 mg/d Silexan or placebo^a^, 10 weeks				
Full analysis set (*n*)	Silexan 104, placebo 108	Silexan 86, placebo 84	Silexan 159, placebo 156	Silexan 103, placebo 102	Silexan 135, placebo 135

Abbreviations: CAS, Covi Anxiety Scale [46]; HAMA, Hamilton Anxiety Rating Scale [19].

a In addition to Silexan 80 mg/d, trial D included treatment groups receiving Silexan 10 or 40 mg/d, and trial E included groups receiving Silexan 160 mg/d or paroxetine. Results for Silexan dosages other than 80 mg/d or for active comparators were not included in our analyses.

### Outcomes and data analyses

Analyses were performed according to a prospectively defined protocol. Outcomes of interest were the total score of the 14-item HAMA as well as the HAMA subscores assessing psychic (items “anxious mood,” “tension,” “fears,” “insomnia,” “intellectual,” “depressed mood,” “behavior at interview”) and somatic (items “somatic complaints, muscular,” “somatic complaints, sensory,” “cardiovascular symptoms,” “respiratory symptoms,” “gastrointestinal symptoms,” “genitourinary symptoms,” “autonomic symptoms”) anxiety [37]. Moreover, we performed analyses on all individual HAMA items as well as on items 1 (“severity of illness”) and 2 (“global improvement”) of the Clinical Global Impressions (CGI) scale [38].

According to Hamilton [19], HAMA total scores between 18 and 24 points indicate mild to moderate anxiety, while scores between 25 and 30 points indicate moderate to severe anxiety. Assuming the cut-off values between mild and moderate as well as between moderate and severe anxiety to be located toward the middle of the proposed categories, we defined mild anxiety as HAMA <22 points, moderate anxiety as HAMA between 22 and 27 points, and severe anxiety as HAMA >27 points for the purpose of our analyses.

In trials A, B, D, and E, post-baseline outcome assessments were performed every 2 weeks while patients of Trial C were assessed at the end of weeks 1, 2, 4, 7, and 10. For longitudinal analyses, the data of trial C of the first week of treatment were not included, and the data of week 7 were analyzed together with the week 8 data of other trials. Moreover, for the calculation of HAMA total score, missing single items were replaced by the mean of the non-missing items of the same patient and visit during longitudinal analyses. The imputation of missing single items was only performed when at least 50% of the items were non-missing.

### Statistical methods

Statistical methods were chosen following the approach described in Hieronymus et al. [13], who performed an item-based, patient-level, post hoc analysis to investigate the association between baseline disease severity and efficacy in the treatment of depression with SSRIs.

The interaction between baseline severity (as a continuous variable) and treatment was assessed using an analysis of covariance (ANCOVA) model with the HAMA total score at week 10 as the dependent variable, treatment as well as trial as fixed factors, the baseline HAMA total score as a covariate, and the interaction between baseline HAMA total score and treatment. Moreover, for the analysis of the HAMA total score, subscores, and individual items between baseline and the end of randomized treatment, longitudinal analyses were performed using mixed models for repeated measures (MMRM). The models included fixed factors for treatment, time (week), trial, and categorized baseline severity. All two-way interactions between baseline severity, treatment, and time, as well as the three-way interaction between baseline severity, treatment, and time were included. Correlations between repeated measures were modeled using unstructured variance/covariance matrices determined separately for each baseline severity category. Kenward-Roger approximation was applied to estimate denominator degrees of freedom. The outcome of interest was the estimated difference between the adjusted means for Silexan and placebo at the end of the treatment (week 10) within the categories defined by baseline severity. In addition, the corresponding standard errors of means (SEM), 95% confidence intervals (CIs), and *p*-values for the differences between the adjusted means were calculated.

Sensitivity analyses included the calculation of standardized mean value differences (Cohen’s d) based on the raw (unadjusted) mean value differences and standard deviations of the observed outcomes at week 10 within the baseline severity categories as well as analysis of variance (ANOVA) models analogous to the MMRMs, but with the HAMA total score at week 10 as the dependent variable (observed case analysis).

For the CGI, we performed ANOVA models as described above, using CGI Item 1 at week 10, absolute change of CGI Item 1 between baseline and week 10, as well as CGI Item 2 at week 10 as dependent variables.

Further sensitivity analyses were performed using the same statistical procedures as described above, but with different HAMA severity classes (baseline total score: <21 vs. 21–27 vs. >27 points; <22 vs. 22–28 vs. >28 point) as well as including only studies performed in patients with subsyndromal anxiety disorders (studies A, B, C).

In this post-hoc analysis, all reported *p*-values are intended to be interpreted exploratively.

SAS version 9.4 on Microsoft Windows 10 was used for all calculations.

## Results

### Study participants and baseline severity of anxiety

The analysis population consisted of 1,172 patients (Silexan, n = 587; placebo, n = 585), representing the full analysis sets of the five clinical trials that were available for analysis. Basic patient characteristics are summarized in Table 2. Further details are included in Bartova et al. [36] and in the original publications referred to in Table 1.

**Table 2.** Study population baseline characteristics (number and percentage or mean ± SD)

Trial	Treatment	Randomized	FAS	Females^a^	Age (years)^a^	HAMA total score^a^
A	Silexan	110	104	73.1%	45.6 ± 11.4	26.8 ± 5.4
	Placebo	111	108	76.9%	46.6 ± 11.3	27.1 ± 5.2
B	Silexan	86	86	72.1%	48.0 ± 11.3	25.5 ± 6.0
	Placebo	84	84	71.4%	46.9 ± 12.7	26.5 ± 6.1
C	Silexan	160	159	66.0%	47.7 ± 12.6	25.7 ± 5.6
	Placebo	158	156	72.4%	47.9 ± 12.6	25.7 ± 5.2
D	Silexan	118	103	76.7%	43.3 ± 11.7	24.6 ± 4.4
	Placebo	113	102	65.7%	45.5 ± 11.5	25.7 ± 5.1
E	Silexan	136	135	70.4%	45.7 ± 11.5	25.8 ± 4.8
	Placebo	137	135	73.3%	44.6 ± 12.3	25.1 ± 4.7
Pooled	Silexan	610	587	71.0%	46.1 ± 11.9	25.7 ± 5.3
	Placebo	603	585	72.1%	46.4 ± 12.2	25.9 ± 5.2

Abbreviations: FAS, full analysis set; HAMA, Hamilton Anxiety Rating Scale.

a Based on full analysis set.

**Figure 1.**
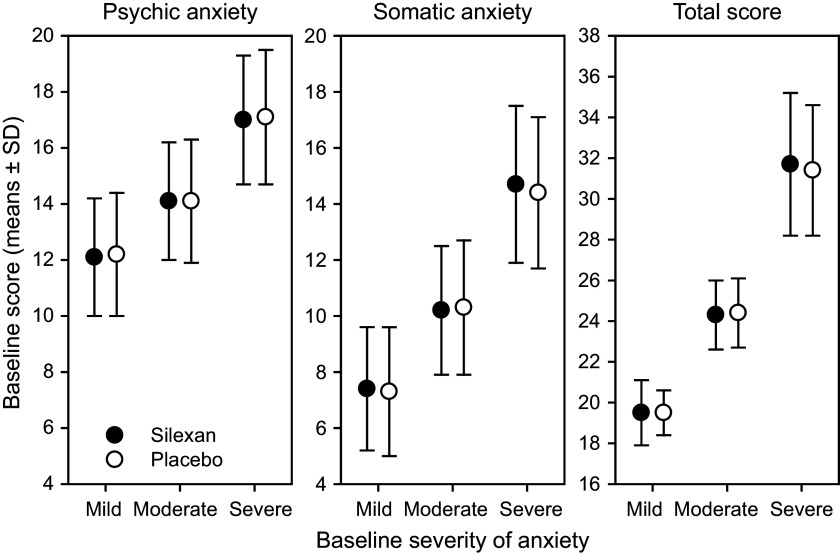
Hamilton Anxiety Rating Scale subscores and total score at baseline, by baseline severity classes (means and standard deviations, full analysis set, pooled data from all trials).

At baseline, 275 patients (Silexan, n = 137; placebo, n = 138) suffered from mild (HAMA total score <22), 475 (Silexan, n = 250; placebo, n = 225) from moderate (HAMA total score 22–27), and 422 (Silexan, n = 200; placebo, n = 222) from severe symptoms of anxiety (HAMA total score >27). Within all subsets, defined by baseline severity, HAMA total scores and subscores were comparable between Silexan and placebo. Figure 1 also shows that in all subsets, baseline scores for psychic anxiety were consistently higher than those for somatic anxiety (note that there are seven HAMA items in each subscore).

For the pooled dataset, including all subjects treated with Silexan or placebo, Table 3 presents the HAMA baseline scores within the baseline severity subsets on an individual item basis, along with the proportion of patients exhibiting each symptom (i.e., having a score ≥1). Most of the difference between patients with mild, moderate, or severe anxiety at baseline was attributable to symptoms assigned to the somatic anxiety subscale. Compared to the mild group, somatic scores for moderate and severe baseline anxiety were 39% and 96% higher, respectively. In comparison, the psychic anxiety scores were only 16% and 39% higher.

**Table 3.** Baseline symptom prevalence by baseline severity of anxiety (full analysis set, pooled data from all trials)

Item/score	Baseline severity of anxiety					
	Mild (*n* = 275)		Moderate (*n* = 475)		Severe (*n* = 422)	
	Mean ± SD	% who scored ≥1	Mean ± SD	% who scored ≥1	Mean ± SD	% who scored ≥1
Total score	19.5 ± 1.4	NA	24.4 ± 1.7	NA	31.5 ± 3.3	NA
Psychic anxiety	12.2 ± 2.2	NA	14.1 ± 2.2	NA	17.0 ± 2.4	NA
Anxious mood	2.3 ± 0.6	100%	2.8 ± 0.6	100%	3.1 ± 0.6	100%
Tension	2.4 ± 0.6	100%	2.8 ± 0.6	100%	3.2 ± 0.6	100%
Fear	0.9 ± 1	60%	1.2 ± 1	60%	1.5 ± 1.2	77%
Insomnia	2.3 ± 1.1	92%	2.6 ± 1	92%	3.1 ± 0.8	100%
Intellectual impairment	1.4 ± 0.8	88%	1.7 ± 0.9	88%	2.3 ± 0.9	96%
Depressed mood	1.5 ± 0.8	88%	1.7 ± 0.9	88%	2.1 ± 1	97%
Behavior at interview	1.2 ± 0.7	83%	1.3 ± 0.8	83%	1.7 ± 0.8	96%
Somatic anxiety	7.4 ± 2.2	NA	10.3 ± 2.4	NA	14.5 ± 2.7	NA
Somatic complaints – muscular	1.3 ± 0.9	79%	1.8 ± 0.9	79%	2.5 ± 0.9	98%
Somatic complaints – sensory	1.2 ± 0.9	75%	1.6 ± 0.9	75%	2.3 ± 0.9	97%
Cardiovascular symptoms	1.1 ± 0.9	65%	1.6 ± 1	65%	2.3 ± 1.1	94%
Respiratory symptoms	0.7 ± 0.8	48%	1.1 ± 0.9	48%	1.8 ± 1.1	86%
Gastrointestinal symptoms	1 ± 0.9	68%	1.4 ± 1	68%	1.8 ± 1.2	81%
Genitourinary symptoms	0.7 ± 0.8	52%	1.1 ± 0.9	52%	1.6 ± 1.1	81%
Autonomic symptoms	1.4 ± 0.8	85%	1.7 ± 0.8	85%	2.2 ± 0.8	98%

Abbreviation: NA, not applicable.

HAMA items with particularly large baseline differences between subsets were respiratory symptoms (+57% and +157% for moderate and severe baseline anxiety, compared to mildly anxious patients), followed by genitourinary symptoms (+57% and +129%), and cardiovascular symptoms (+45% and +109%). All of these belong to the somatic anxiety subscale. Baseline values for those treated with Silexan compared to those treated with placebo were similar (data not shown).

**Figure 2.**
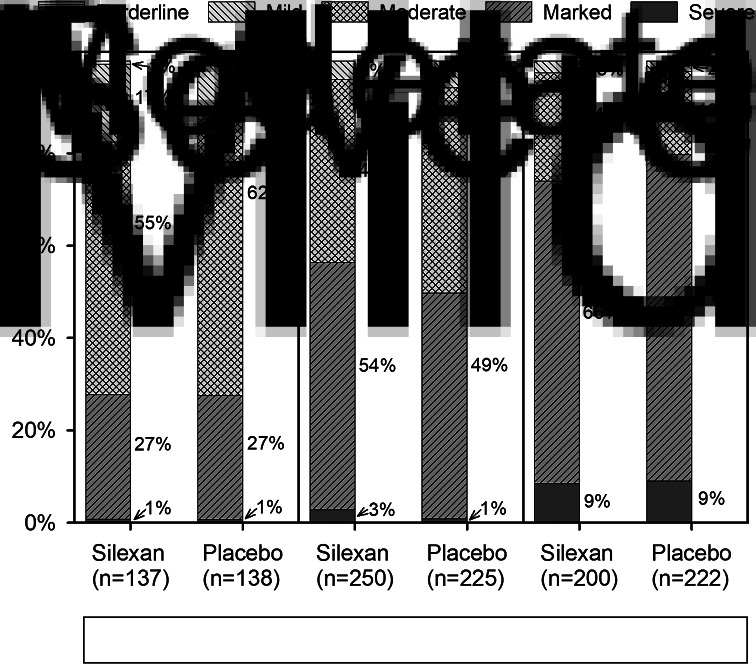
Baseline assessment of Clinical Global Impressions item “severity of illness,” by Hamilton Anxiety Rating Scale total score at baseline (proportions within subsets, full analysis set, pooled data from all trials).

According to CGI Item 1, most patients of all subsets were at least moderately mentally ill at baseline (Figure 2), regardless of their HAMA total score. Broken down by baseline anxiety subset, the corresponding proportions (≥ moderate) were 86% (236/275) for mild, 95% (452/475) for moderate, and 98% (412/422) for severe anxiety symptoms.

### Treatment effects in patients with mild, moderate, and severe anxiety symptoms

In patients treated with Silexan, severity at baseline was shown to positively influence the extent of improvement (interaction between treatment and HAMA total score at baseline: *p* < 0.0001; ANCOVA with factors for treatment and trial, HAMA total score at baseline as covariate, and interaction between treatment and HAMA total score at baseline). Placebo-treated patients with higher HAMA baseline scores tended to show above-average scores at the end of treatment. This was particularly true for patients treated with placebo with baseline scores from about 25 points where average treatment end scores increased around 15 points to about 25 points. In contrast, patients in the Silexan group achieved at treatment end mean scores ranging around 12 points, almost regardless of their baseline score.

**Figure 3.**
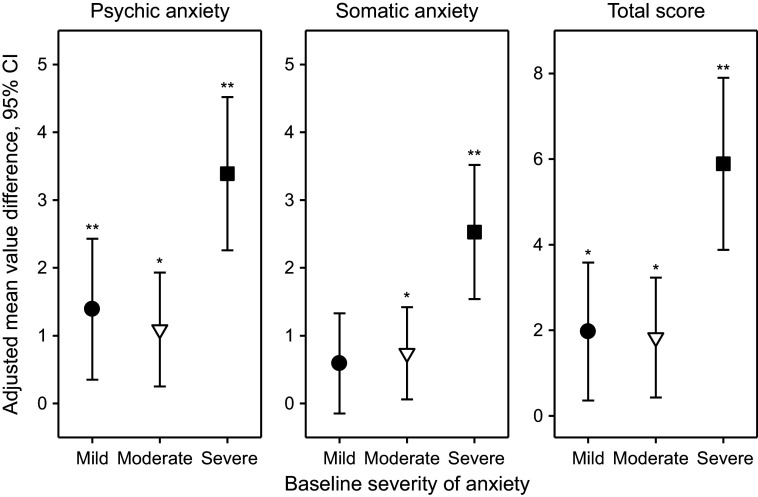
Adjusted mean value differences between Silexan and placebo for the Hamilton Anxiety Rating Scale subscores and total score at week 10, by baseline severity classes (with 95% confidence intervals, *p < 0.05; **p = 0.01; full analysis set, pooled data from all trials).

For the HAMA psychic and somatic anxiety subscores and total score, Figure 3 shows the adjusted mean value differences between Silexan and placebo at week 10 (higher values favor Silexan), by symptom severity at baseline. Except for the somatic anxiety subscore in patients with initially mild anxiety, Silexan was significantly superior to placebo in reducing psychic, somatic, and total symptom severity in all subsets defined by baseline anxiety. Independent of baseline severity, treatment effects were consistently more pronounced on psychic anxiety than on somatic anxiety symptoms. Moreover, while Silexan treatment effects were of similar magnitude in patients with mild or moderate baseline anxiety, they were substantially more pronounced in those with severe anxiety symptoms at baseline.

**Figure 4.**
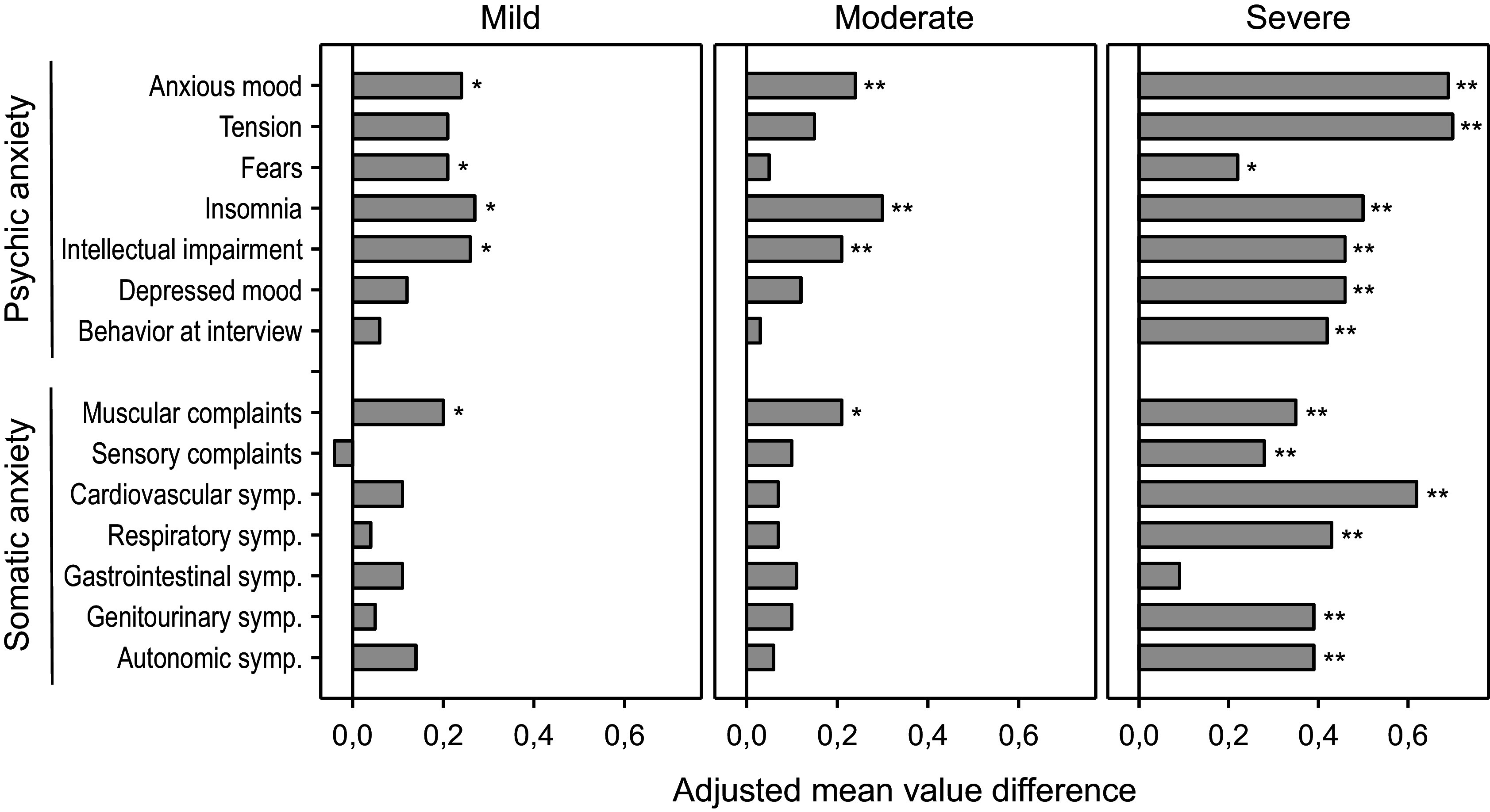
Adjusted mean value differences between Silexan and placebo for the Hamilton Anxiety Rating Scale single item scores at week 10, by baseline severity classes (*p < 0.05; **p = 0.01; full analysis set, pooled data from all trials).

A HAMA item-based analysis (Figure 4) showed that on average all of them improved under treatment with Silexan except for item sensory complaints in patients with mild baseline anxiety. Severely ill patients showed significant improvements for all HAMA items except gastrointestinal symptoms. In comparison, patients with mild or moderate baseline symptoms exhibited significant treatment effects mainly for symptoms from the psychic anxiety subscore. For the mild and moderate subsets, items that showed a significant treatment effect included three (mild anxiety subset: anxious mood, insomnia, intellectual impairment) and four (moderate anxiety subset: anxious mood, insomnia, muscular complaints, intellectual impairment) out of the top five items (items with highest average impairment scores at baseline) (Table 3).

For CGI Item 2 (global improvement compared to baseline), the adjusted treatment group mean value differences of 0.54 (95% confidence interval: 0.25; 0.82) points, 0.32 (0.10; 0.54) points, and 0.77 (0.52; 1.01) points favoring Silexan were observed for patients with mild, moderate, and severe baseline symptom severities, respectively (*p* < 0.001 for all comparisons).

We performed sensitivity analyses using alternative cut-offs between mild, moderate, and severe baseline anxiety and performed ANOVA models with only the end-of-treatment value of the outcome of interest as the dependent variable instead of MMRMs over the entire time course, as well as analyses that included only patients from trials A–C with subsyndromal anxiety disorders (see Methods for details). The results of these sensitivity analyses were consistent with those of the main analyses described above (data not shown).

## Discussion

Only a few studies investigated the association between the severity of anxiety symptoms and the efficacy of anxiolytic psychopharmacotherapy. However, those studies only focused on treatment with antidepressants, notably SSRIs and SNRIs. Even if this medication class is recommended to manage/treat anxiety disorders, other compounds with a different mechanism of action (such as Silexan) can be also regarded as evidence-based treatment options in anxiety disorders [2, 7–11]. With regard to medicinal herbs, Silexan achieved in a meta-analysis the highest effect size in terms of efficacy when analyzing the herbs with an at least moderate cumulative sample size [39].

Here we present the first analysis to investigate the association between initial severity of anxiety and the efficacy of Silexan. To our knowledge, this is also the first pooled analysis examining the association between baseline anxiety symptom severity and treatment efficacy that is both item-based and based on individual patient data.

Our analyses showed a clear association between the initial severity of anxiety symptoms and the magnitude of treatment effect of Silexan in comparison to placebo. The main results are thus consistent with the work of de Vries et al. [18], who showed that initial severity moderated the efficacy of treatment with SSRIs and SNRIs in patients with GAD. In our study, the association was evident both on patient level (treatment effects tended to be larger in patients with higher baseline severity) and on individual symptom level (symptoms with higher average baseline severity tended to show larger differences between Silexan and placebo).

Patient subsets (defined by severity) tended to show a more intense baseline symptom burden for the psychic HAMA subscore than for the somatic subscore. Nonetheless, the larger part of the overall differences between patients with mild, moderate, or severe baseline anxiety was driven by differences in somatic symptoms. In patients with mild baseline anxiety, four out of the five HAMA items with the lowest average baseline scores were from the somatic anxiety subscore, and 10 out of the 14 HAMA items – among them all items of the somatic anxiety subscore – had a baseline mean score of less than 1.5 points.

For items with average baseline scores in the range of 1.5 points or less, room for improvement is limited (“floor effect”), and it is thus not surprising that no significant treatment effect of Silexan over placebo could be shown for such items. Accordingly, the observation that treatment effect sizes in patients with mild or moderate anxiety were notably lower than those in patients with severe anxiety might be at least partly explained by the sparsity of somatic symptoms at baseline in these subgroups compared to the severe anxiety subgroup.

These results show that patients with mild or moderate anxiety nevertheless benefit substantially from treatment with Silexan. The severity-defined subsets exhibited significant treatment effects of Silexan over placebo for the HAMA total score as well as for psychic and somatic (moderate and severe baseline anxiety only) anxiety. Moreover, improvement was found to be highly consistent across the individual HAMA items. Patients with mild or moderate baseline severity mainly improved in the items such as anxious mood, insomnia, intellectual impairment, and muscular complaints (the only HAMA item from the somatic domain). Those items are among the principal diagnostic criteria of GAD according to DSM-5 [40] and ICD-11 [41]. Interestingly, a recently published HAMA factor analysis [42] has found muscular complaints to be more closely associated with the “cognitive” factor identified during the analysis than with the “physiological” factor. This is especially interesting as muscular complaints were among the items that scored appreciably high at baseline in our dataset and showed significant improvement over placebo in mildly and moderately anxious patients during treatment with Silexan.

Our findings with respect to the different HAMA items provide a clinical description of typical patients who presumably benefit most from a pharmacotherapy with Silexan. These are preferentially patients with anxious mood, tension, insomnia, intellectual impairment, muscular complaints, and – in general – more severely ill patients.

Results for the CGI show that not only patients with severe anxiety but also those with mild or moderate anxiety showed global improvements beyond the amelioration of specific, individual symptoms. This is consistent with the observation that patients with subsyndromal forms of anxiety disorder meet some but not all of the diagnostic thresholds of GAD (i.e., number and/or duration of symptoms), but nevertheless show significant suffering and therefore require appropriate treatment [25, 43]. Compared to the HAMA results, the CGI results also indicate a certain lack of sensitivity of the HAMA total score for mapping improvement in patients whose baseline impairment is low for some of the symptoms assessed, due to the “floor effect” described above. Psychiatric symptom scales such as the HAMA typically possess a favorable sensitivity to change when all or at least most of the symptoms assessed show appreciable levels of baseline impairment. Lower absolute overall improvement in patients whose baseline intensity of some of the symptoms is low may thus not necessarily be indicative of lack of benefit from treatment, but rather may be due to a lack of sensitivity of the symptom scale in this subpopulation. In our study, the CGI, which is not a symptom scale but which assesses impairment and improvement globally, was apparently not affected by a comparable “floor effect” and may thus have been more sensitive to treatment effects in patients with a narrower symptom spectrum.

The observed associations between baseline severity and the magnitude of the anxiolytic treatment effect were based on placebo-controlled trials with Silexan and can therefore not be generalized to other anxiolytic psychopharmacotherapy. It should be noted, however, that our results for the HAMA total score are consistent with an individual patient data meta-analysis of de Vries et al. [18]. In this work, GAD patients treated with SSRIs and SNRIs were examined, even though the authors did not examine HAMA subscores or individual items.

Our work is limited by the absence of a universally accepted classification for the severity levels of anxiety symptoms. Therefore, our categorization of trial participants into subsets of mild, moderate, or severe baseline anxiety was based on an extrapolation of the “mild to moderate” and “moderate to severe” subsets as proposed by Hamilton [19] in the original HAMA publication. Our sensitivity analyses, with slight variations of the cut-offs between the subsets, produced results that did not differ substantially from our main analysis and led to the same conclusions. However, this is admittedly not the only justifiable severity classification. Of note, some authors have suggested lower cut-offs than 22 points for separating mild from moderate anxiety [e.g., 44, 45], already classifying patients with a total score >7 points as mildly anxious. Patients with such low HAMA total scores could not be included in our analysis as for all trials the minimum HAMA total score for inclusion was 18 points, an often-chosen cut-off.

Our analyses were based on the HAMA and CGI scales that can be regarded as primarily efficacy-related outcomes. It should be therefore considered that our findings are not necessarily accompanied by similar effects in measurement evaluating explicitly quality of life.

A further limitation arises from the fact that all analyzed trials were performed by the manufacturer of Silexan. However, our systematic literature search did not identify any other relevant studies.

In conclusion, using the HAMA total score as an outcome, we found a tendency toward smaller treatment effects of Silexan in patients with mild to moderate baseline anxiety. This can, at least in part, be explained by the absence or low intensity of some HAMA-assessed symptoms at baseline, thus leaving only minimal room for improvement (“floor effect”). In this regard, the CGI, which showed significant improvements for Silexan over placebo in all baseline severity subsets, could be a more suitable scale, due to a global rather than a symptom-dependent assessment improvement. Overall, the analyses show that patients with mild or moderate baseline anxiety also benefit substantially, and treatment with Silexan thus is justified and indicated in patients suffering from an anxiety disorder at all severity levels.

## Footnotes

1 Silexan^®^ is a proprietary lavender oil of Dr. Willmar Schwabe GmbH & Co. KG, Karlsruhe, Germany.
